# Big Data Blind Separation

**DOI:** 10.3390/e20030150

**Published:** 2018-02-27

**Authors:** Mujahid N. Syed

**Affiliations:** Department of Systems Engineering, King Fahd University of Petroleum & Minerals, Dhahran 31261, Saudi Arabia; smujahid@kfupm.edu.sa or

**Keywords:** big data, blind signal separation, locally dominant sources, correntropy ranking

## Abstract

Data or signal separation is one of the critical areas of data analysis. In this work, the problem of non-negative data separation is considered. The problem can be briefly described as follows: given $X\in R^{m\times N}$, find $A\in R^{m\times n}$ and $S\in R_{+}^{n\times N}$ such that X=AS. Specifically, the problem with sparse locally dominant sources is addressed in this work. Although the problem is well studied in the literature, a test to validate the locally dominant assumption is not yet available. In addition to that, the typical approaches available in the literature sequentially extract the elements of the mixing matrix. In this work, a mathematical modeling-based approach is presented that can simultaneously validate the assumption, and separate the given mixture data. In addition to that, a correntropy-based measure is proposed to reduce the model size. The approach presented in this paper is suitable for big data separation. Numerical experiments are conducted to illustrate the performance and validity of the proposed approach.

## 1. Introduction

Transforming data into information is a key research direction of the current scientific age. During the data collection phase, it is often the case that the data cannot be collected from the actual data generating locations (sources). Typically, a nearby physically connected location (station) that is accessible can be used for the data collection. If the station is influenced by more than one source, then the data collected at the station provides mixed information from the multiple sources (mixture data). This creates a challenging problem of identifying the source data from the given mixture data. Such a problem is typically known as a data (or signal) separation problem.

In this paper, a linear mixing type data separation problem is considered. The generative model of the problem in its standard form can be written as:

(1) X=AS,

where $X\in R^{m\times N}$ denotes the given mixture matrix, $A\in R^{m\times n}$ is the unknown mixing matrix, and $S\in R^{n\times N}$ denotes the unknown source matrix. The problem can be further classified into overdetermined (*m* > *n*), undetermined (*m* < *n*), and square or determined (*m* = *n*) cases. The overdetermined case can be transformed into the square case by using the Principal Component Analysis (PCA) method [1,2]. The undetermined cases often result in loss of information or redundancy in the representation. Usually, approximate recovery is done based on the prior probability assumptions. Typically, Gaussian or Laplacian priors are used to estimate the mixing matrix. The idea is to identify the directions of maximum density via clustering. These directions correspond to the identification of the mixing matrix [3,4]. Once the mixing matrix is estimated, the source matrix is obtained by solving series of least square problems. In this paper, the data separation problem for m≥n cases will be considered.

In addition to that, the above problem given in Equation (1) can be solved under numerous assumptions based on the level of available information. In this work, both A and S are assumed to be unknown. In such a scenario, the above signal separation problem is known as the “Blind” Signal Separation (BSS) problem. Seminal ideas of the BSS problem can be found in [5], where the authors illustrated the idea of BSS via an example of two source signals (*n* = 2) and two mixture signals (*m* = 2). Their objective was to recover source signals from the mixture signals, without any further information. However, it has been known since then that the recovery of sources from a linear mixture is imperfect, and pragmatic recovery needs various identifiability conditions. In [6], various approaches and the identifiable conditions of the BSS problem are summarized.

One of the well known traditional solution approaches to the BSS problem is the Independent Component Analysis (ICA) [7]. The ICA-based approaches are built upon the key assumption of the statistical independence among the rows of S. The basic objective of ICA is to separate the input data into statically independent components. During the last three decades, there have been a large amount of papers on signal analysis that are devoted to the usage of ICA. These approaches are designed not only for the linear but also for the nonlinear signal mixing scenarios. They have also been designed for the overdetermined, undetermined, as well as square cases of the BSS problem. Some of the well known approaches in ICA are based on mutual information [8]; negentropy [9]; projection pursuit [10]; infomax [11]. Recent applications of ICA include bio-medical data analysis [12,13,14,15,16,17]; power system analysis [18], and audio and speech [19]. It is out of the scope of this paper to summarize all the typical approaches of ICA. Therefore, interested readers are referred to [7,20] (and the references therein). One of the major critiques pertaining to ICA is the existence of independent sources. Some of the signal analysis areas (such as biomedical and hyperspectral image analysis) may not satisfy the independence criterion (see [21]). Thus, alternative approaches to ICA for BSS have also been a critical research direction in the area of signal analysis.

Other prominent approaches of BSS, apart from ICA, assume some sparsity or geometric structure in S [22]. In this work, the focus is on the sparsity-based approaches of extracting non-negative sources. This area is widely known as Sparse Component Analysis (SCA) [23]. Specifically, following are the basic assumptions that are considered while solving the SCA problem.

**Basic SCA assumptions:**

1. Every column of the source matrix is non-negative.
2. Source matrix has a full row rank.
3. Mixing matrix has a full column rank, and m≥n.
4. The rows of the source matrix, and columns of the mixing matrix have unit norm.
5. Source matrix is sparse.

The first assumption provides a mathematical advantage in designing the solution algorithms. Basically, the non-negativity assumption transforms the BSS problem into a convex programming problem [24,25,26]. In addition to that, non-negative source signals are very common in sound and image analysis. The next two assumptions ensure that the problem is recoverable (solvable). The fourth assumption is perhaps the limit of all BSS approaches, which is related to scalability and uniqueness (see [27]). The fifth assumption is the key sparsity assumption of the SCA-related approaches. Different scenarios of the SCA problem arise with different structures of the sparsity. The typical structures of sparsity discussed in the SCA literature can be classified as:

- **Locally Dominant Case:** In addition to the basic assumptions, for a given row *r* of S, there exists at least one unique column *c* such that:
  (2) $$s_{i,c}\left\{\begin{array}{cc} >0 & \mathrm{if}\;i=r\\ =0 & \mathrm{otherwise} \end{array}\right.$$
- **Locally Latent Case:** In addition to the basic assumptions, for a given row *r* of S, there exists at least (n−1) linearly independent and unique columns $C_{r}$ such that:
  (3) $$s_{i,c}\left\{\begin{array}{cc} =0 & \mathrm{if}\;i=r\;\&\;c\;\in \;C_{r}\\ >0 & \mathrm{if}\;i\neq r\;\&\;c\;\in \;C_{r} \end{array}\right.$$
- **General Sparse Case:** This is the default case.

The first case is one of the widely known cases in the SCA literature (see [28,29] for recent literature reviews). The second case is new to the of SCA literature, and few recent papers address this case [30]. The first two cases have identifiability conditions, which assure identification and recovery of the source signals. If the conditions of the first two cases do not apply to the given data, then the SCA problem belongs to the last general case. Typically, the general case may not have perfect identification (apart from the scalability and uniqueness issues). For the general case, minimum volume-based approaches [26,31,32], and extreme direction-based approaches [33] have been proposed to approximately recover the source matrix. Some special cases of sparse structure, apart from the above cases, that can recover original source data have also been studied in the literature, for example see [34].

**Figure 1:**
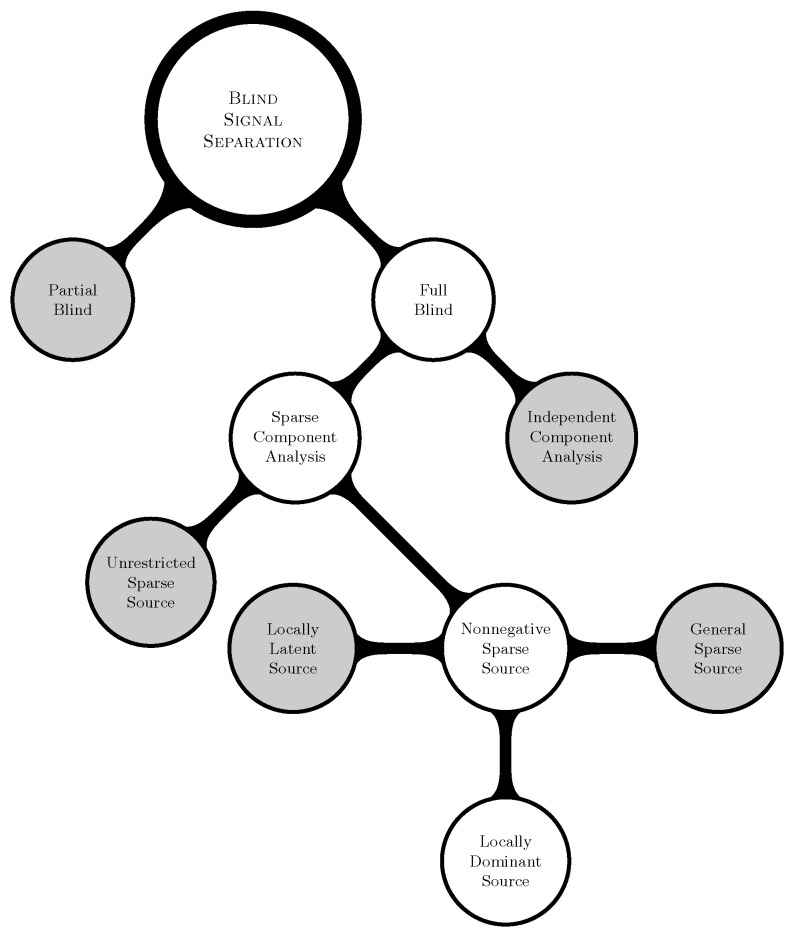
Overview of the “Blind” Signal Separation (BSS) problem.

In addition to the above, the conditions on X that improve the separability of sources are studied in the literature [35,36]. Methods that exploit spectral variability can be seen in [37]. Time series (frequency and transformation analysis) based methods to identify sparse sources have been presented in [4,38]. Further methods developed on the assumption of SCA can be found in [39,40]. The prominent application areas of SCA include, but are not limited to, the following: Blind Hyperspectral Unmixing (BHU) [41], chemical analysis [42], Nuclear Magnetic Resonance (NMR) spectroscopy [43], etc. Figure 1 portrays various linear BSS methods available in the literature. The grayed areas in the figure represent the research areas that will not be considered further in this paper. Thus, the branches unrelated to the paper that emerge from the grayed areas are ignored in the figure.

One of the critical gaps found in the SCA literature is the unavailability of a method to test the existence of a locally dominant assumption from the mixture data (X). In this paper, a novel mathematical programming and correntropy-based approach is presented for testing the locally dominant source assumption. The proposed approach also provides a solution for the locally dominant SCA problem.

Throughout this paper, the following notation styles are used. A capital letter bold face character, like B, indicates a matrix. A small letter bold face character with or without a subscript, like $b_{r}$, indicates the *r*th column vector of matrix B. A small letter bold face character with a special subscript, like $b_{p•}$, indicates the transpose of the *p*th row vector of matrix B. A non-bold small letter character, like $b_{p,r}$, represents the *p*th row *r*th column element of matrix B.

The rest of the paper is organized as follows: Section 2 introduces the locally dominant case. Specifically, it displays the existing formulations from the literature, and presents the proposed novel formulation. A correntropy-based ranking method to eliminate the non-extreme data points is developed in Section 3. By incorporating the proposed model and the proposed ranking method, a tailored solution approach for the big data separation problem is developed in Section 4. A numerical study to assert the performance of the proposed approach is illustrated in Section 5. Finally, the paper is concluded with discussions in Section 6.

## 2. Locally Dominant Case

Consider the following determined or square version of the SCA model:

(4) X=AS.

Each column $x_{i}$ for i=1,…,N of the mixture matrix (X) can be represented as follows:

(5) $$x_{i}=\sum_{j=1}^{n}s_{j,i}a_{j}\;\forall \;i\;=\;1,\ldots ,N,$$

where $a_{j}$ is the *j*th column of the mixing matrix (A), and $s_{j,i}$ is the *j*th row *i*th column element of the source matrix (S). Equation (5) highlights that every column vector of X is a linear combination of the column vectors of A. Since the source matrix (S) is non-negative (i.e., $s_{j,i}\geq 0$ for all *i* & *j*), the combination is a conic combination. Thus, the columns of X are spanned by the columns of A. In other words, the extreme column vectors of X are the columns of A. Therefore, the locally dominant case boils down to the identification of the extreme vectors of X [24].

If all the columns of X are non-negative, then normalizing every column of X with respect to Norm-1 makes the columns of X coplanar. That is, all the columns are contained in the following lower dimensional plane:

(6) $$\sum_{j=1}^{n}x_{j,i}=1\;\forall \;i\;=\;1,\ldots ,N.$$

Now, the extreme points of X on the lower dimensional plane correspond to the columns of A. In addition to that, if some of the elements of X are negative, then the columns of X are projected onto a suitable lower dimensional plane. There are many approaches in the literature that are designed to work on this lower dimensional plane (affine hull) [25,44]. The advantage of working on this plane is that the extreme vector columns of X will form the vertices of a lower dimensional simplex. Thus, identifying the extreme points will result in the identification of the mixing matrix. Next, a few well known mathematical formulations and solution approaches for SCA from the literature are presented.

### 2.1. Conventional Formulations

One of the earliest mathematical formulations that identifies the extreme vectors of X is proposed in [24]. The idea is to pick one column of X, say $x_{c}$, and check the possibility of it being an extreme vector. The formulation corresponding to $x_{c}$ is given as follows:

(7) $$\begin{array}{cc} min.: & \\ & \;{∥\sum_{\begin{array}{c} i=1\\ i\neq c \end{array}}^{N}\alpha_{i}x_{i}-x_{c}∥}_{2}\\ s.t.: & \end{array}$$

(8) $$\begin{array}{c} \;\alpha_{i}\;\geq \;0\;\forall \;i=1,\ldots ,N,\;\&\;i\neq c, \end{array}$$

where $\alpha_{i}\;\geq \;0$, ∈R is the variable that corresponds to the weight of $x_{i}\in X$ for i=1,…,N. The key idea that is exploited in the formulation is that the extreme vectors cannot be represented by a non-negative weighted combination of the other data vectors. The above formulation is a least square minimization problem. In the worse case, the formulation has to be executed *N* times for each $x_{c}$, i.e., for c=1,…,N. In the best case, the formulation has to be executed *n* times. A nonzero value of the objective function indicates that the vector $x_{c}$ is an extreme vector of X.

Another approach, called Convex Analysis of Mixtures of Non-negative Sources (CAMNS), that works on the affine hull is presented in [25]. The solution approach of CAMNS involves two major steps. In the first step, the parameters $C\in R^{N\times (n-1)}$ and $d\in R^{N}$ of the affine hull are estimated as follows:

(9) $$\begin{array}{c} d=\frac{1}{N}\sum_{j=1}^{N}x_{j•}\; \end{array}$$

(10) $$\begin{array}{c} U=X^{T}-d\; \end{array}$$

(11) $$\begin{array}{c} C={\left[eigv\left(UU^{T}\right)\right]}_{1,\ldots ,(n-1)} \end{array}$$

where $x_{j•}$ is the *j*th row of X. In Equation (10), vector d is subtracted from all the rows of X, and in Equation (11), matrix C contains the columns corresponding to the eigenvectors associated with the largest (n−1) eigenvalues of $UU^{T}$. Basically, this first step is similar to the dimensionality reduction process executed in the PCA. In the second step, the following mathematical model is repeatedly solved:

(12) $$\begin{array}{cc} max/min: & \\ & r^{T}\left(C\alpha +d\right)\\ s.t.: & \end{array}$$

(13) $$\begin{array}{c} \;c_{i•}^{T}\alpha +d_{i}\geq 0\;\forall \;i=1,\ldots ,N \end{array}$$

where $r\in R^{N}$ is a generated vector, $c_{i•}$ is an *i*th row of C, and $\alpha \in R^{\left(n-1\right)}$ is the unknown variable. The above formulation exploits the notion that the optimal solution of a linear program exists at the extreme points. For a given r, the formulation is solved twice; one time as the maximization problem and the other time as the minimization problem. This is done in order to get one (or maybe two) new extreme point(s) in every iteration. The (n−1) extreme points are identified by resolving the above formulation repeatedly with respect to different r vectors. The crux of this method is hidden in the generation of r and convergence of the approach, which are the main concepts presented in [25].

Notice that the above two ideologies extract the columns of A sequentially. In addition to the above approaches, typical approaches in the literature identify the columns of A sequentially [28,29,45]. Therefore, there is no mechanism to validate the locally dominant assumption from X. One of the main objectives of this paper is to build such a mechanism using a mathematical formulation-based approach. In the following subsection, a novel formulation is presented that can provide the above mechanism.

### 2.2. Envelope Formulation

In this section, a mathematical model that can simultaneously identify all the extreme vectors of X under the locally dominant assumption is proposed. To the best of our knowledge, an exact method that identifies all the extreme vectors in a single shot is unavailable.

Let $X_{2}$ be the data obtained after normalizing each column of X with respect to Norm-2. Let $y_{i}\in X_{2}$ be the *i*th column of $X_{2}$. Let $a^{T}y=b$ be a plane that is inclined in such a way that all the columns of $X_{2}$ are contained in one halfspace of the hyperplane, and the origin is in the other halfspace. Such a hyperplane is referred to as linear envelope in this work. The envelope can be written as:

(14) $$a^{T}y_{i}\geq b\;\forall \;i=1,\ldots ,N,$$

where $a\in R^{m}$ corresponds to the normal vector of the envelope, and b≥0 is a constant. Out of infinite possible representations of the above envelope, an envelope with b=1 is selected for further analysis. Now, the distance between any vector $y_{i}$ and the envelope can be written as:

(15) $$\begin{array}{cc} d(y_{i}) & =\frac{|a^{T}y_{i}-1|}{{\left||a|\right|}_{2}} \end{array}$$

(16) $$\begin{array}{c} \;=\frac{a^{T}y_{i}-1}{{\left||a|\right|}_{2}} \end{array}$$

(17) $$\begin{array}{c} \;\propto a^{T}y_{i}-1=p\left(y_{i}\right)\;\forall \;i=1,\ldots ,N. \end{array}$$

Equation (16) follows from Equation (14). Ignoring the denominator (${\left||a|\right|}_{2}$) in Equation (16) results in a proportional or scaled distance ($p(y_{i})$). Among infinite possible linear envelopes, the aim is to find the tightest or supporting envelope. A formulation that can identify the tightest envelope is given as follows:

(18) $$\begin{array}{cc} min.: & \\ & \sum_{i=1}^{N}\left(a^{T}y_{i}-1\right)\\ s.t.: & \end{array}$$

(19) $$\begin{array}{c} \;a^{T}y_{i}\geq 1\;\forall \;i=1,\ldots ,N, \end{array}$$

where $a\in R^{m}$ is the unknown variable. The above formulation can be equivalently written as:

(20) $$\underset{-}{\mathrm{Formulation}}:\;$$

(21) $$\begin{array}{cc} min.: & \\ & \mu^{T}a\\ s.t.: & \end{array}$$

(22) $$\begin{array}{c} \;y_{i}^{T}a\geq 1\;\forall \;i=1,\ldots ,N, \end{array}$$

where $\mu \in R^{m}$ is defined as $\mu =\sum_{i=1}^{N}y_{i}$. It is assumed that the duplicate and/or all zero columns of $X_{2}$ are removed before the execution of the above model. The aim of the above model is to find the envelope that has the minimum distance with respect to all the columns of $X_{2}$. The above formulation is linear, and needs to be executed only once to identify all the extreme vectors. That is, at the optimal solution, the data points ($y_{i}$’s) corresponding to the active constraints in Equation (19) will correspond to the extreme vectors of $X_{2}$. It can be seen that the above formulation is always feasible. Furthermore, due to the design of the constraint given in Equation (19), the problem is linear. That is, the design allowed the usage of proportional distance (Equation (17)) instead of the nonlinear actual distance (Equation (15)).

In addition to that, from the LP theory, only *m* constraints will be active at the optimal solution. Let Θ be the matrix containing the columns of X corresponding to the *m* active constraints. Identifying the columns of A requires the following additional steps: Calculate $q_{i}=\Theta^{-1}x_{i}$ for i=1,…,N. If $q_{i}\geq 0$ for i=1,…,N, then Θ corresponds to A. However, if any element of $q_{i}$ is strictly less than 0 for any i=1,…,N, then it indicates that the locally dominant assumption is invalid for the given X. Thus, this serves as a test for the existence of the locally dominant assumption.

The above test works for typical data mixing without noise. However, the image data is usually integer data. There is always rounding, taking place at the source or mixture level. Therefore, the rounding effect needs to be incorporated into the above condition. It is proposed in this paper to follow the heuristic method to incorporate the rounding effect. Let $\nu =0.5|\Theta^{-1}e|$, where $e\in R^{n}$ is a vector of all ones. The notion is that the rounding will create a maximum error of ±0.5 in each pixel element. Thus, the maximum error in any pixel element will be strictly less than 0.5. Therefore, the check for image data will be as follows: If $q_{i}+\nu \geq 0$ for i=1,…,N, then Θ corresponds to A.

Furthermore, the above idea can be extended for mixing scenarios containing noise. For instance, a level of tolerance can be used to analyze the noisy mixture data. Let ψ≥0, $\in R^{m}$ be a tolerance parameter selected by the user. Then, based on the earlier discussion, the check for noisy data will be as follows: If $q_{i}+\psi \geq 0$ for i=1,…,N, then Θ corresponds to A. The precise value of ψ may not be available for a given scenario. Hence, the parameter will be empirically selected based on trial experiments. In Section 5, an experiment that highlights the usage of parameter ψ for noisy data is illustrated.

## 3. Point Correntropy

Correntropy is a generalized correlation measure based on the concept of entropy. It is typically used in detecting local similarity between two random variables. Roughly speaking, it is a kernelized version of the conventional correlation measure. The measure first appeared in [46,47], and its usage as a cost function was illustrated in [48,49,50,51,52]. The optimization properties of the cost function are presented in [53]. The correntropy cost function (or the correntropic loss) for *N* errors is defined as:

(23) $$F\left(\varepsilon \right)=\beta (1-\frac{1}{N}\sum_{i=1}^{N}k\left(\varepsilon_{i},\sigma \right)),$$

where $\beta ={\left[1-e^{\left(\frac{-1}{2\sigma^{2}}\right)}\right]}^{-1}$ is a scaling parameter, $\varepsilon \in R^{N}$ is an array of errors, and k() is the transformation kernel function with parameter σ. In this work, a Gaussian kernel is selected, i.e., $k\left(\varepsilon ,\sigma \right)=e^{\left(\frac{-\varepsilon^{2}}{2\sigma^{2}}\right)}$. Equation (23) is readily separable with respect to sample errors, and can be rewritten as:

(24) $$F\left(\varepsilon \right)=\sum_{i=1}^{N}f\left(\varepsilon_{i}\right)$$

where $f\left(\varepsilon_{i}\right)=\frac{\beta }{N}\left(1-k\left(\varepsilon_{i},\sigma \right)\right)$, and it will be referred to as point estimate of the correntropic loss.

Let $\varepsilon_{i}$ be an error corresponding to the *i*th column vector of X. For a given kernel parameter σ, the point estimate provides similarity information of the *i*th vector with respect to the other data vectors of X. Based on the geometry of the vectors, the extreme vectors’ similarity with respect to the central vectors should be typically less than the other non-extreme vectors of X. Thus, the point estimate of the correntropic loss function can be used as a measure to differentiate extreme and non-extreme vectors of X.

## 4. Solution Methodology

**Figure 2:**
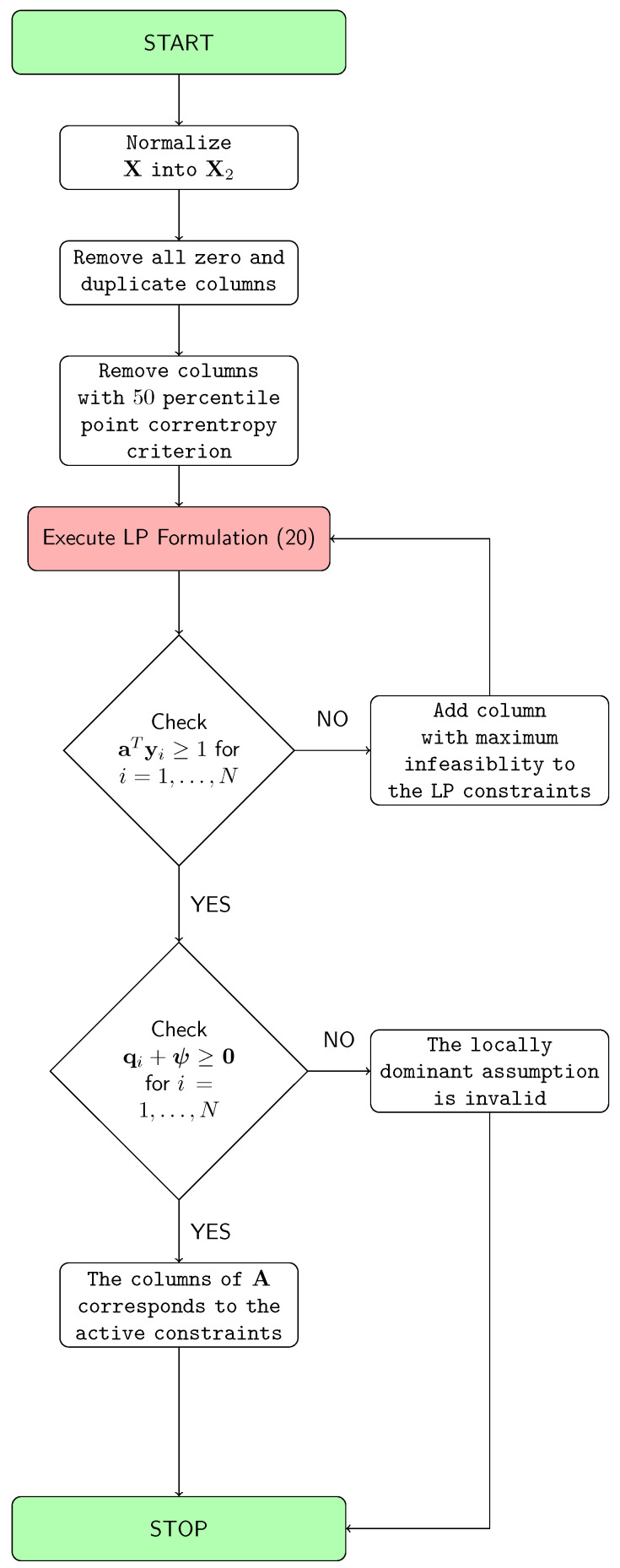
The proposed approach.

Our goal is to develop a geometric separation method for the non-negative data mixing problem, that can be applied to the “Big Data” scenarios. The concepts developed in Section 2 and Section 3 are tailored with respect to big data, and the following solution approach is proposed. The summary of the proposed approach is illustrated in Figure 2 and Algorithm 1.

**Algorithm 1:** The Proposed Algorithm. **Data**: Given $X\in R^{m\times N}$ **Result**: Find $A\in R^{m\times n}$ and $S\in R_{+}^{n\times N}$ such that X=AS $X_{2}$ = normalize(X); Remove all zero columns and duplicate columns from $X_{2}$, and say $X_{2}\in R^{m\times \hat{N}}$; Estimate σ from S; Obtain $X_{R}$ by removing all columns with the 50 percentile point correntropy criterion from $X_{2}$; Let $X_{E}=X_{2}\backslash X_{R}$; Let $y_{i}$ be the *i*th column of $X_{2}$; a = Solution of LP Formulation (20) with respect to data $X_{R}$; **while**
$a^{T}y_{i}<1\;for\;i=1,\ldots ,N$
**do** 
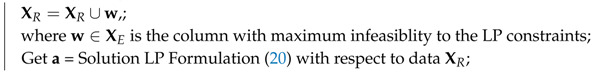
 **end** Let Θ be the matrix containing the columns of X corresponding to the active constraints at the optimal solution of Formulation (20); Calculate $q_{i}=\Theta^{-1}x_{i}$ for i=1,…,N; Set ψ equal to 0 for non-noisy non-image data mixing, equal to ν for non-noisy image data mixing, or equal to the user-specified value for noisy mixing.; **if**
$q_{i}+\psi \geq 0\;\forall i=1,\ldots ,N$
**then** 
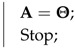
 **else** 
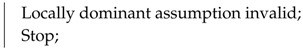
 **end**

**Data Ranking:** As seen earlier, the extreme vectors of $X_{2}$ contain all the relevant information that is needed for separation (i.e., identifying A and S). Other data vectors are redundant in identifying the mixing matrix. Thus, the point estimate of the correntropic loss can be evaluated at all the data points with respect to the central columns of $X_{2}$. Those data points that have low value of the correntropic loss can be removed from the data set $X_{2}$ without losing any information. The major issues in implementing the above idea are as follows: how to select the right value for σ, and how to define $\varepsilon_{i}$ corresponding to $y_{i}$ for i=1,…,N.

The value of σ represents the kernel width, and should be large enough to contain the central vectors. However, it should be small enough to exclude the extreme vectors. In the following, we propose a practical method to estimate the value of σ. Let S be a sample of columns randomly selected from $X_{2}$. Let $\delta_{j}=\max_{\begin{array}{c} \forall \;i1,i2\in S\\ i1<i2 \end{array}}\left\{y_{j,i1}-y_{j,i2}\right\}$, where $y_{j,i1}$ is the *j*th element of $y_{i1}$. Based on the trial experiments, we found that $\sigma =\frac{\sum_{j=1}^{n}\delta_{j}}{\sqrt{2n}}$ is a good choice for the kernel width. Furthermore, the value of c should correspond to the center of the columns of $X_{2}$. An approximate estimate for c can be $c=\frac{g+h}{2}$, where $g_{j}=\max_{j}\left\{y_{j,i}\right\}$, $h_{j}=\min_{j}\left\{y_{j,i}\right\}$ for i∈S. Thus, simple (and practical for big data) estimate of error for $y_{i}$ will be $\varepsilon_{i}=y_{i}-c$ for i=1,…,N. Based on the trial experiments, it can be concluded that the larger the size of S, the better the estimation. Furthermore, the strategy to eliminate the columns from the trial experiments is as follows. All the columns of $X_{2}$ that have the point estimate value lower than 50 percentile are removed from further consideration.

**Handling a Large Number of Constraints:** From Formulation (20), it can be seen that the big data corresponds to a large number of constraints. However, only *m* constraints are active, and the rest of the constraints are redundant (i.e., the rest of the constraints will never be active). The proposed data ranking method eliminates a good amount of the redundant constraints, depending upon the distribution of columns in the data set.

Let $X_{R}\subseteq X_{2}$ be the data matrix obtained after eliminating possible central vectors, and let $X_{E}\subset X_{2}$ be the eliminated central vectors. If the columns of $X_{2}$ are reorganized, then there exists a partition such that $X_{2}=\left[X_{R}|X_{E}\right]$. Once the tightest envelope is obtained for $X_{R}$ by solving Formulation (20), the envelope is validated with respect to $X_{E}$. If all the columns of $X_{E}$ fall in the same half space (i.e., Equation (14) is feasible with respect to $X_{2}$), then it can be guaranteed that none of the extreme vectors of $X_{2}$ were eliminated. However, if there is any infeasibility detected, then the column of $X_{E}$ with maximum infeasibility is added to the LP, and the LP is resolved.

**Performance Index:** In order to evaluate the performance of the proposed approach, distance-based metrics will be used. Specifically, two metrics (one for the mixing matrix, and the other for the source matrix) will be used in this work. The following error measure is for the mixing matrix:

(25) $$e_{A}=\frac{1}{n^{2}}\sum_{j=1}^{n}{∥\frac{a_{j}}{\left|\right|a_{j}\left|\right|}-\frac{{\hat{a}}_{\left[j\right]}}{\left|\right|{\hat{a}}_{\left[j\right]}\left|\right|}∥}_{2}$$

where $a_{j}$ is the *j*th column of the original mixing matrix A, and ${\hat{a}}_{\left[j\right]}$ is the corresponding column to $a_{j}$, obtained from the recovered mixing matrix $\hat{A}$. The corresponding columns are identified by the Hungarian algorithm [54]. The source matrix is obtained as follows:

(26) $$\hat{S}=\underset{S\in R_{+}^{n\times N}}{argmin}\{∥X-\hat{A}S{∥}_{2}\}$$

The above approach can be replaced by $\hat{S}={\hat{A}}^{-1}X$, whenever ${\hat{A}}^{-1}$ exists. Similar to $e_{A}$, the following error measure is for the source matrix:

(27) $$e_{S}=\frac{1}{nN}\sum_{j=1}^{n}{∥\frac{s_{j•}}{\left|\right|s_{j•}\left|\right|}-\frac{{\hat{s}}_{\left[j•\right]}}{\left|\right|{\hat{s}}_{\left[j•\right]}\left|\right|}∥}_{2}$$

where $s_{j•}$ is the *j*th row of the original source matrix S, and ${\hat{s}}_{\left[j•\right]}$ is the corresponding row to $s_{j•}$, obtained from the recovered source matrix $\hat{S}$.

## 5. Numerical Experiments

In order to illustrate the performance of the proposed approach, numerical experiments are presented. The experiments are divided into four groups. In the first group of experiments, simulated non-noisy data is used to test the performance and sensitivity of the proposed approach. In the second group of experiments, image data mixtures are used to test the applicability of the proposed approach on real image data. The third and fourth group of experiments compare the proposed approach with the well known SCA methods in the literature. In all the instances of this section, the following specifications were used: S={1,…,N}. All the random mixing matrices contain columns with unit norm. The LP resolving step is skipped in order to identify the number of instances in which the extreme vectors were eliminated. The LP was solved via the dual simplex method, using the state of the art Cplex 12.0 solver [55]. All the instances were solved on an Intel Xeon 2.4 GHz workstation, with 16 logical processors and 32 GB of RAM.

### 5.1. Simulated Data Separation

**Setup:** Given *n* and *N*, a random source matrix S that satisfies the locally dominant assumption is generated. For the generated S matrix, 100 random mixing matrices A are generated. Then, using the X=AS equation, 100 random mixture matrices are generated. On each mixture matrix, the proposed approach is implemented. This study is executed for all the following combinations of **n** =5,7,9 and 11, and **N** = 1000, 5000, 10,000, 50,000, 100,000, 500,000 and 1,000,000. In addition to that, this study is also executed for **n** =10,20,40,60,80 and 100 when **N** = 1,000,000. The value of ψ is set to zero in this experiment.

**Figure 3:**
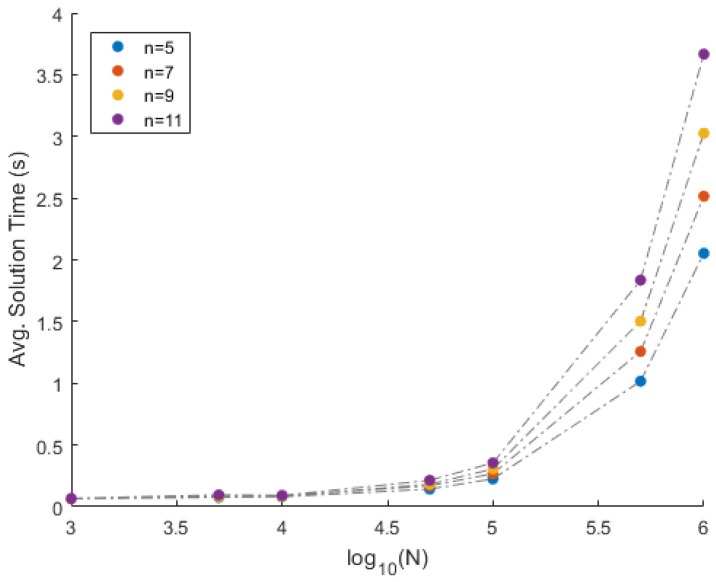
Time VS $\log_{10}\left(N\right)$ comparison on the simulated data.

**Figure 4:**
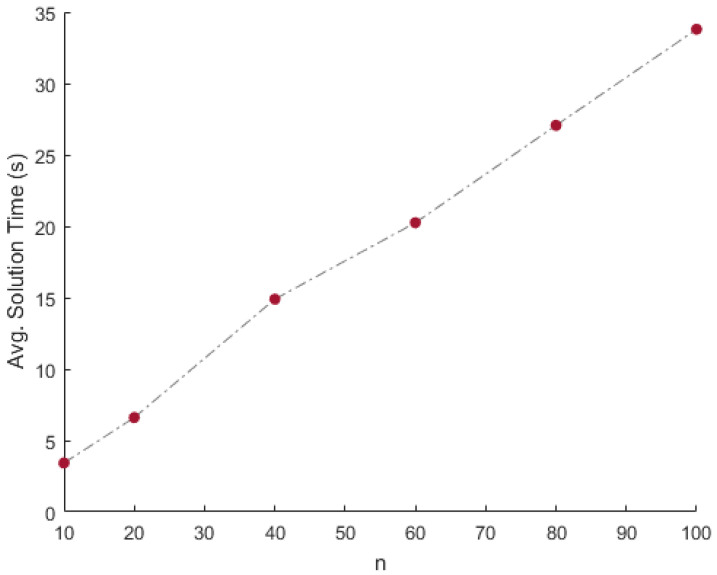
Time vs. *n* comparison on the simulated data.

**Results:** Using one mixture matrix as input, and using the proposed approach, matrices $\hat{A}$ and $\hat{S}$ are recovered. This experiment is repeated 100 times for a given combination of *n* and *N*. The performance of the proposed approach is displayed in Table 1. The column corresponding to **mErrA** (**vErrA**) indicates the mean (variance) of error $e_{A}$ over the 100 instances. Similarly, columns **mErrS** and **vErrS** correspond to the mean and variance of error $e_{S}$ respectively. The column corresponding to **mTime** (**vTime**) indicates the mean (variance) of the solution time per instance in seconds (milliseconds) over the 100 instances. In addition to that, the column corresponding to **mRed** (**vRed**) indicates the mean (variance) of the percentage of columns eliminated over the 100 instances. Finally, the column corresponding to **nMiss** indicates the number of times the extreme vectors were eliminated based on the 50 percentile criterion. Since the mixtures are clean (i.e., no noise is added to the mixture data), the recovery is perfect. This can be seen from the very low average error (**mErrA** and **mErrS**) over the 100 iterations. Furthermore, the method is consistent in the recovery of the matrices, and it can be justified from the low variance in the error (**vErrA** and **vErrS**). The suitability and applicability of the proposed approach to big data can be seen from the solution time. For instance, Figure 3 and Figure 4 illustrate the average time in seconds required to solve one instance of the proposed approach for *N* data points and *n* data sources. The behavior of the solution time with respect to $\log_{10}\left(N\right)$ is exponential. In other words, the solution time increases linearly with respect to *N*. Furthermore, from Figure 4, it can be observed that the solution time is linear with respect to *n*. Thus, the algorithm is suitable for big data scenarios. Moreover, the 50 percentile criterion removes exactly 50% of the data points in all the cases, with zero variance. This is due to the fact that the source matrices are uniformly randomly generated. Due to the uniform generation of the source matrices, none of the extreme vectors were eliminated.

### 5.2. Image Mixture Separation

**Setup:** In the following experiments, image data available from the literature and online repositories are considered (see Table 2). Each source image of an image set is reshaped into one row vector. Then, the reshaped images are row-wise stacked together to generate the S matrix. The source matrices are pre-processed in order to satisfy the locally dominant assumption. Next, for each source matrix S, 100 random A matrices are generated, and correspondingly 100 random X matrices are analyzed using the proposed approach. Table 2 summarizes the details of the image sets that are considered in this subsection. The first column conveys the name of the image set that is being considered. The column corresponding to **n** indicates the total number of sources, and the column corresponding to **N** indicates the total data points (or column vectors) in X. The value of ψ is set to ν in this experiment.

**Figure 5:**
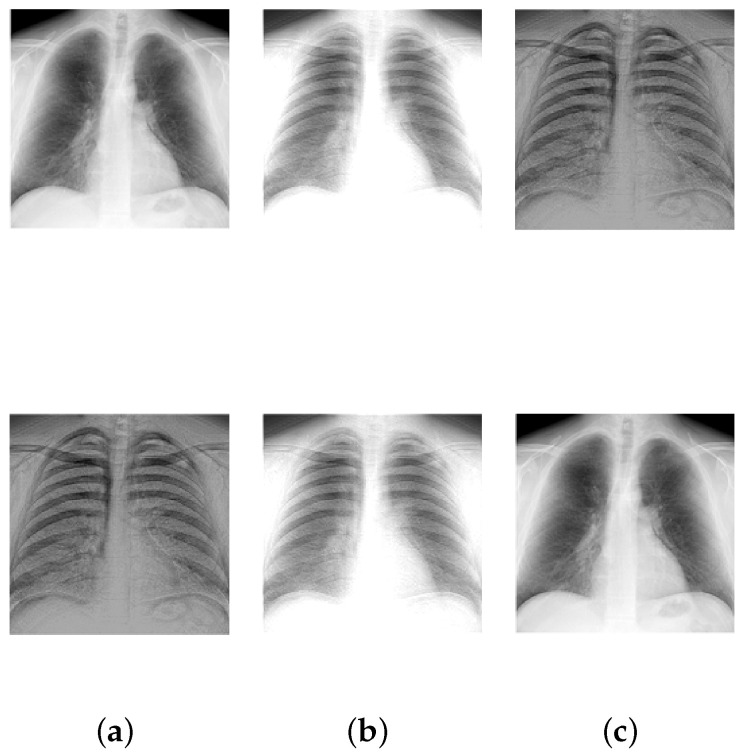
Mixing and unmixing of chest X-rays. (**a**) Original; (**b**) Mixture; (**c**) Recovered.

**Figure 6:**
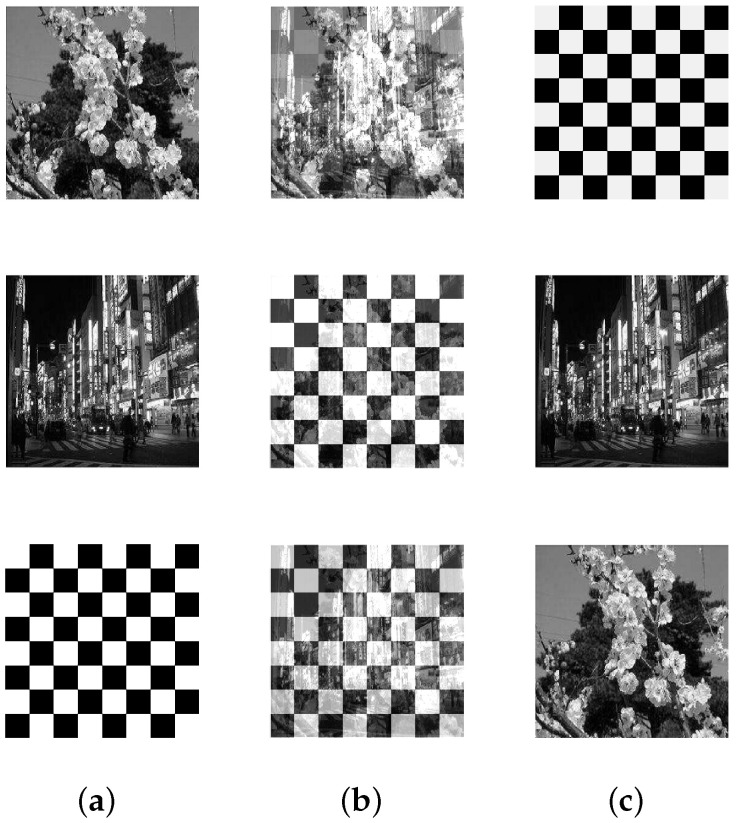
Mixing and unmixing of scenery. (**a**) Original; (**b**) Mixture; (**c**) Recovered.

**Figure 7:**
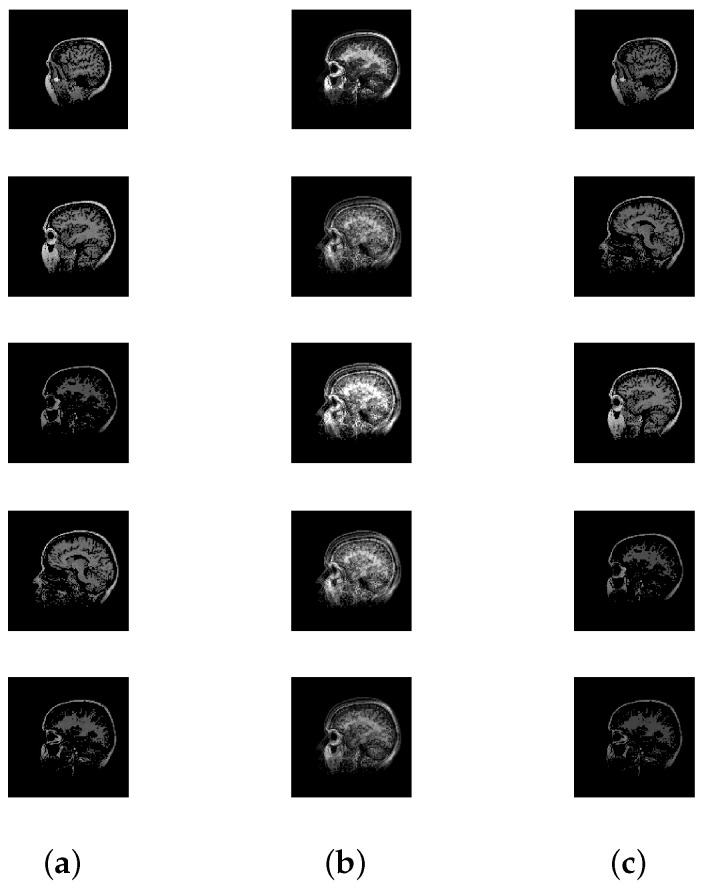
Mixing and unmixing of a CT scan. (**a**) Original; (**b**) Mixture; (**c**) Recovered.

**Figure 8:**
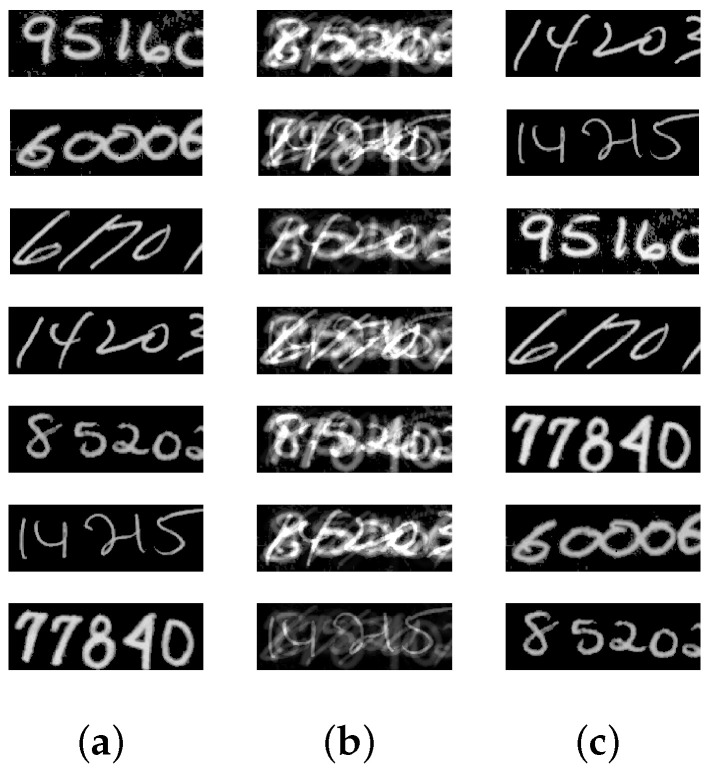
Mixing and unmixing of zip codes. (**a**) Original; (**b**) Mixture; (**c**) Recovered.

**Figure 9:**
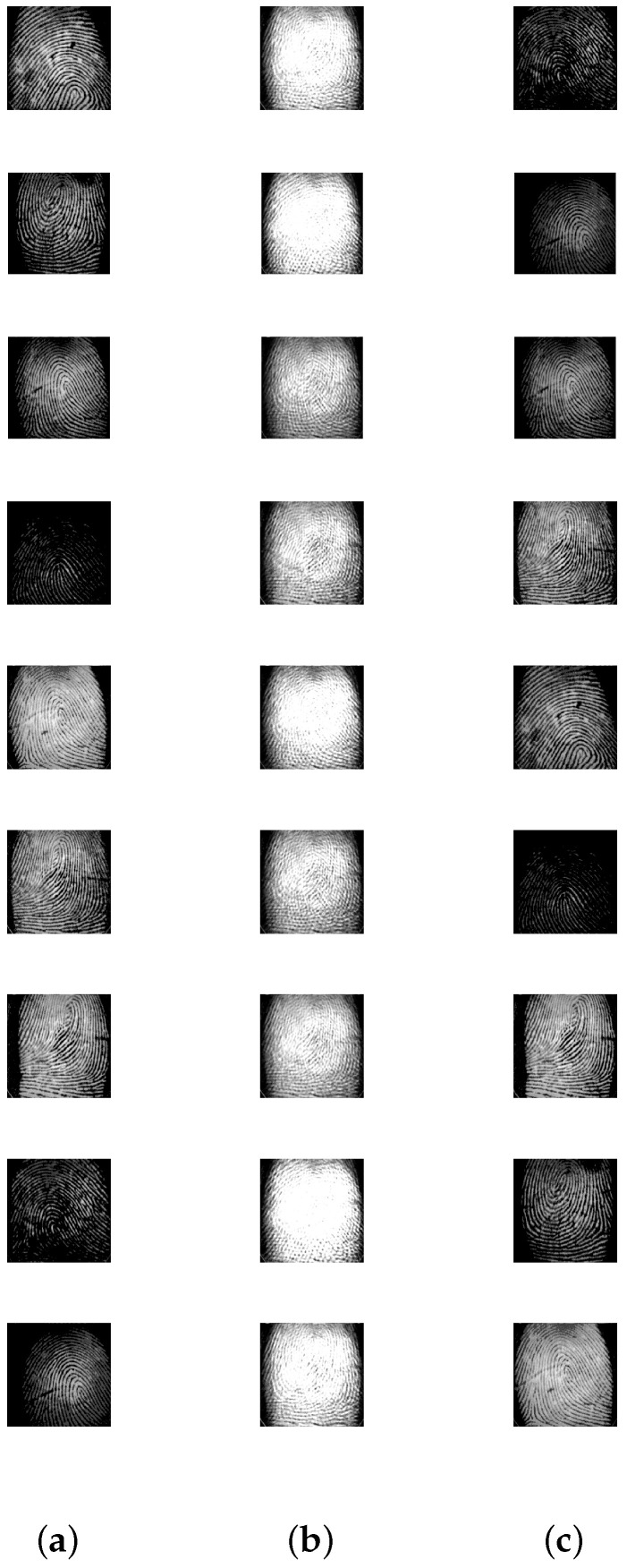
Mixing and unmixing of a finger print. (**a**) Original; (**b**) Mixture; (**c**) Recovered.

**Results:** Table 3 displays the results after executing the proposed approach on the 100 mixture instances of each image set. The columns have the notation similar to the earlier experiment, except that the **vTime** column units are in seconds. Moreover, Figure 5, Figure 6, Figure 7, Figure 8 and Figure 9 depict the results. Low mean errors ($e_{A}$ and $e_{S}$) over the 100 runs are obtained for all the image sets. This shows that the method precisely recovers A and S matrices. A low value in the corresponding variance column indicates the high level of consistency of the proposed approach. The solution time, specifically for the finger print data set, indicates the applicability of the proposed approach for big data with complex image mixing scenarios. Based on the results, it can be seen that the 50 percentile criterion eliminates a good amount (more than 50%) of the redundant columns. However, in some instances (at most 7 percentage in one instance), the criterion eliminated some of the extreme vectors.

### 5.3. Comparative Experiment-I

**Setup:** Given *n* and *N*, a random source matrix S that does not satisfy the locally dominant assumption is generated in this experiment. For the generated S matrix, 100 random mixing matrices A are generated. Then, using the X=AS equation, 100 random mixture matrices are generated. This study is executed for **n** =5,7,9,11,13 and 15, and for **N** = 10,000. The value of ψ is set to zero in this experiment. Well known methods from the SCA literature are compared with the proposed approach. The methods that are used for the comparison are N-FINDR ([44]), VCA ([45]), MVSA ([56]). The objective of this experiment is to highlight that the above three (like the other typical algorithms in the literature) do not have the capability to test the locally dominant assumption from the knowledge of X. To the best of my knowledge, only an exhaustive search similar to the one presented in [24] can do such a test. However, the proposed approach does not require such an exhaustive search.

**Results:** Table 4 displays the results after executing the proposed and selected approaches on the 100 randomly generated mixture instances. The column corresponding to **mErrA** (**vErrA**) indicates the mean (variance) of error $e_{A}$ over the 100 instances for the three methods used from the literature. The lines in the column corresponding to **ErrA** indicate that the proposed approach was unable to identify any mixing matrix. The reason for the lines is the non-existence of the locally dominant assumption in the source data. This information is captured in the column corresponding to **TnMiss**. The numbers in the **TnMiss** column indicate the total number of times the proposed approach exited with the “no locally dominant sources” token. Based on the results, it can be seen that the other algorithms try to find the best match for the columns of A. However, they are unable to validate the locally dominant assumption. This is due to the fact that no such test is available in the literature. However, in all the scenarios, the proposed approach was able to conclude that the input data is not a mixture of sources that contain the locally dominant signals.

### 5.4. Comparative Experiment-II

**Setup:** In this experiment, a random source matrix S that satisfies the locally dominant assumption, is generated. For the generated S matrix, 100 random mixing matrices A are generated. Then, using the X=AS equation, 100 random mixture matrices are generated. In each mixture matrix, 5% of the columns are randomly selected, and a uniform noise between 0 and 0.01 is added to all the elements of the selected columns. This study is executed for **n** =5,7,9,11,13 and 15, and for **N** = 10,000. Well known methods from the SCA literature are compared with the proposed approach. The methods that are used for the comparison are N-FINDR ([44]), VCA ([45]), MVSA ([56]). The objective of this experiment is to comparatively assess the performance of the proposed and selected methods in noisy data. In this experiment, the value of ψ is defined as follows: $\psi =\rho |\Theta^{-1}e|$, where $e\in R^{n}$ is a vector of all ones, and ρ takes the following values: 0,0.2,0.4,…,1.

**Figure 10:**
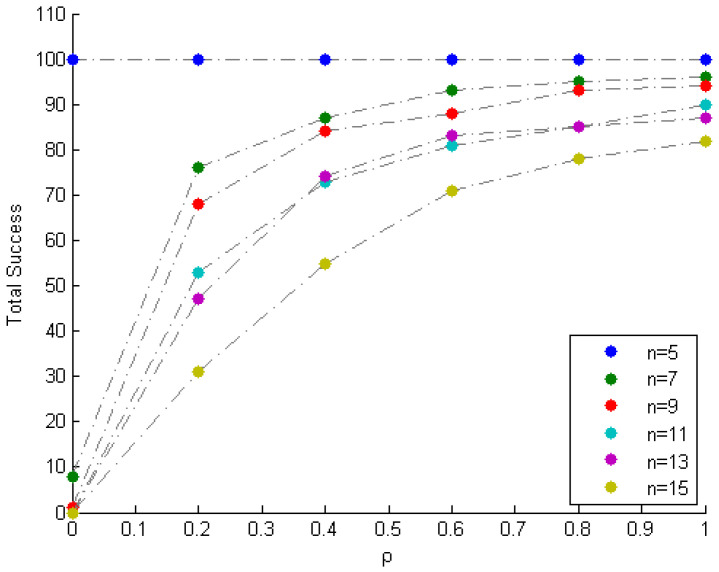
Total success vs. ρ (the tolerance parameter).

**Results:** Table 5 displays the results after executing the proposed and selected approaches on the 100 randomly generated noisy mixture instances. The columns corresponding to VCA, MVSA and N-FINDR present the average error $e_{A}$ over the 100 instances. The proposed approach is executed 100 times for each value of ρ, and the average error $e_{A}$ for each value of ρ is archived. The column corresponding to the proposed approach presents the best of the average errors $e_{A}$ over the values of ρ. Table 6 (Figure 10) indicates the total number of times the method fails (succeeds) to identify the mixing matrix, for various values of ρ. Based on the low value of error in the proposed column, and the trends depicted in Figure 10, it can be seen that the proposed approach recovers A and S in the majority of the noisy instances for higher values of ρ. Moreover, as *n* increases, the complexity of mixing increases, and thus the proposed approach requires a higher value of ρ for the recovery of A and S.

## 6. Discussion and Conclusions

The SCA approaches are relatively new to the BSS problem when compared to the ICA approaches. The main critique that often appears with respect to locally dominant SCA approaches is the validity of the locally dominant criterion. In this paper, a mathematical modeling-based approach is proposed that can validate the existence of the locally dominant criterion from the given mixture matrix. That is, the formulation can be used not only to identify the mixing matrix, but also to validate the assumption presented in Equation (2). Although the approach is proposed for the determined case, it can also be applied to the overdetermined cases. The columns of the matrix X are proportional to the number of constraints in the proposed LP. Thus, big data often leads to LP with many redundant constraints. We propose the usage of interior point methods, when the total number of constraints is very high [57]. Moreover, LP decomposition-based approaches for SCA can also be developed to improve the solution time [58]. In addition to that, the LP presolve theory [59] can be designed to eliminate the redundant constraints in the proposed SCA approach. Roughly speaking, the point correntropic ranking method may be seen as a novel probabilistic approach for removing LP redundant constraints. The proposed method of estimating the point correntropy is computationally cheap, and can be applied to the big data scenarios. From the simulated data study, it can be seen that if the input data is uniformly distributed, then the 50 percentile criterion can be raised to a higher value. However, from the image data set, it is clear that the real world data is rarely uniformly distributed. Thus, the 50 percentile criterion is a good estimate to avoid the LP resolving. From the comparative experiments, it can be concluded that the proposed approach validates the locally dominant assumption in non-noisy and noisy mixing scenarios. To summarize, the proposed approach provides new insights into the BSS problem.

## Figures and Tables

**Table 1:** Performance of the proposed approach on the simulated data.

n × N	mErrA	vErrA	mErrS	vErrS	mTime	vTime	mRed	vRed	nMiss
5 × 1000	1.04 × 10${}^{-17}$	9.78 × 10${}^{-36}$	1.17 × 10${}^{-20}$	4.76 × 10${}^{-42}$	0.0647	0.07095	50	0	0
5 × 5000	1.08 × 10${}^{-17}$	1.16 × 10${}^{-35}$	2.03 × 10${}^{-21}$	5.24 × 10${}^{-43}$	0.0756	0.04114	50	0	0
5 × 10,000	1.07 × 10${}^{-17}$	1.26 × 10${}^{-35}$	9.50 × 10${}^{-22}$	1.88 × 10${}^{-43}$	0.08	0.16436	50	0	0
5 × 50,000	1.10 × 10${}^{-17}$	1.13 × 10${}^{-35}$	5.10 × 10${}^{-22}$	5.39 × 10${}^{-44}$	0.1427	0.09452	50	0	0
5 × 100,000	1.02 × 10${}^{-17}$	8.22 × 10${}^{-36}$	4.85 × 10${}^{-22}$	3.21 × 10${}^{-44}$	0.2254	0.12459	50	0	0
5 × 500,000	1.01 × 10${}^{-17}$	9.28 × 10${}^{-36}$	1.81 × 10${}^{-22}$	6.50 × 10${}^{-45}$	1.0166	2.2	50	0	0
5 × 1,000,000	1.03 × 10${}^{-17}$	1.11 × 10${}^{-35}$	1.19 × 10${}^{-24}$	8.55 × 10${}^{-50}$	2.0526	1.9	50	0	0
7 × 1000	7.28 × 10${}^{-18}$	3.47 × 10${}^{-36}$	1.30 × 10${}^{-20}$	3.41 × 10${}^{-42}$	0.0641	0.05835	50	0	0
7 × 5000	6.85 × 10${}^{-18}$	2.72 × 10${}^{-36}$	1.71 × 10${}^{-21}$	1.08 × 10${}^{-43}$	0.0816	0.06362	50	0	0
7 × 10,000	6.86 × 10${}^{-18}$	2.28 × 10${}^{-36}$	1.01 × 10${}^{-21}$	7.70 × 10${}^{-44}$	0.0891	0.11015	50	0	0
7 × 50,000	7.08 × 10${}^{-18}$	1.89 × 10${}^{-36}$	7.05 × 10${}^{-22}$	4.27 × 10${}^{-44}$	0.1689	0.14786	50	0	0
7 × 100,000	6.78 × 10${}^{-18}$	2.46 × 10${}^{-36}$	1.14 × 10${}^{-22}$	5.67 × 10${}^{-45}$	0.2675	0.14104	50	0	0
7 × 500,000	7.26 × 10${}^{-18}$	2.61 × 10${}^{-36}$	6.11 × 10${}^{-23}$	1.80 × 10${}^{-45}$	1.2584	1.3	50	0	0
7 × 1,000,000	6.85 × 10${}^{-18}$	2.62 × 10${}^{-36}$	9.93 × 10${}^{-23}$	1.77 × 10${}^{-45}$	2.5154	3.3	50	0	0
9 × 1000	5.50 × 10${}^{-18}$	1.05 × 10${}^{-36}$	1.59 × 10${}^{-20}$	2.94 × 10${}^{-42}$	0.067	0.10896	50	0	0
9 × 5000	5.82 × 10${}^{-18}$	1.42 × 10${}^{-36}$	2.44 × 10${}^{-21}$	1.96 × 10${}^{-43}$	0.0812	0.05954	50	0	0
9 × 10,000	5.62 × 10${}^{-18}$	1.30 × 10${}^{-36}$	8.13 × 10${}^{-22}$	4.63 × 10${}^{-44}$	0.0835	0.08635	50	0	0
9 × 50,000	5.52 × 10${}^{-18}$	1.38 × 10${}^{-36}$	2.62 × 10${}^{-22}$	5.69 × 10${}^{-45}$	0.1821	0.12421	50	0	0
9 × 100,000	5.57 × 10${}^{-18}$	1.31 × 10${}^{-36}$	3.79 × 10${}^{-22}$	1.68 × 10${}^{-44}$	0.3066	0.1855	50	0	0
9 × 500,000	5.40 × 10${}^{-18}$	1.36 × 10${}^{-36}$	9.78 × 10${}^{-23}$	1.84 × 10${}^{-45}$	1.5029	1	50	0	0
9 × 1,000,000	5.32 × 10${}^{-18}$	1.21 × 10${}^{-36}$	1.49 × 10${}^{-22}$	2.10 × 10${}^{-45}$	3.0258	3.1	50	0	0
11 × 1000	4.05 × 10${}^{-18}$	4.77 × 10${}^{-37}$	1.75 × 10${}^{-20}$	3.13 × 10${}^{-42}$	0.0672	0.02826	50	0	0
11 × 5000	4.16 × 10${}^{-18}$	5.04 × 10${}^{-37}$	2.03 × 10${}^{-21}$	9.47 × 10${}^{-44}$	0.096	0.10778	50	0	0
11 × 10,000	4.21 × 10${}^{-18}$	4.87 × 10${}^{-37}$	1.31 × 10${}^{-21}$	7.33 × 10${}^{-44}$	0.0916	0.00928	50	0	0
11 × 50,000	4.09 × 10${}^{-18}$	5.02 × 10${}^{-37}$	6.61 × 10${}^{-22}$	2.97 × 10${}^{-44}$	0.2137	0.05729	50	0	0
11 × 100,000	4.12 × 10${}^{-18}$	3.79 × 10${}^{-37}$	1.90 × 10${}^{-22}$	6.92 × 10${}^{-45}$	0.355	0.25758	50	0	0
11 × 500,000	4.14 × 10${}^{-18}$	3.96 × 10${}^{-37}$	1.18 × 10${}^{-22}$	1.66 × 10${}^{-45}$	1.8358	0.69888	50	0	0
11 × 1,000,000	4.21 × 10${}^{-18}$	4.37 × 10${}^{-37}$	8.91 × 10${}^{-23}$	8.11 × 10${}^{-46}$	3.667	5.4	50	0	0
10 × 1,000,000	4.71 × 10${}^{-18}$	7.54 × 10${}^{-37}$	8.46 × 10${}^{-23}$	8.89 × 10${}^{-46}$	3.4245	2.5	50	0	0
20 × 1,000,000	1.83 × 10${}^{-18}$	5.10 × 10${}^{-38}$	1.12 × 10${}^{-22}$	6.32 × 10${}^{-46}$	6.6053	9.3	50	0	0
40 × 1,000,000	7.84 × 10${}^{-19}$	5.33 × 10${}^{-39}$	4.22 × 10${}^{-23}$	9.61 × 10${}^{-47}$	14.8988	64.8	50	0	0
60 × 1,000,000	7.32 × 10${}^{-19}$	4.62 × 10${}^{-38}$	1.05 × 10${}^{-22}$	2.18 × 10${}^{-46}$	20.2509	628.9	50	0	0
80 × 1,000,000	6.51 × 10${}^{-19}$	6.39 × 10${}^{-39}$	7.51 × 10${}^{-23}$	1.40 × 10${}^{-46}$	27.0654	101.7	50	0	0
100 × 1,000,000	5.13 × 10${}^{-19}$	2.83 × 10${}^{-39}$	4.65 × 10${}^{-23}$	5.57 × 10${}^{-47}$	33.7978	136.6	50	0	0

**Table 2:** Image Mixture Separation Data.

Image Set	n	N
Chest X-rays	2	26,896
Scenery	3	65,536
CT Scans	5	16,384
Zip Codes	7	12,672
Finger Print	9	90,000

**Table 3:** Image mixture separation results.

Image Set	mErrA	vErrA	mErrS	vErrS	mTime	vTime	mRed	vRed	nMiss
Chest X-rays	2.45 × 10${}^{-17}$	2.81 × 10${}^{-34}$	1.33 × 10${}^{-22}$	2.02 × 10${}^{-44}$	0.0755	4.89 × 10${}^{-5}$	81.632	0.0101	0
Scenery	2.06 × 10${}^{-17}$	5.30 × 10${}^{-35}$	4.10 × 10${}^{-22}$	4.12 × 10${}^{-44}$	0.109	9.81 × 10${}^{-5}$	73.2417	0.0033	7
CT Scan	1.19 × 10${}^{-17}$	9.09 × 10${}^{-36}$	4.80 × 10${}^{-22}$	1.20 × 10${}^{-44}$	0.0679	1.34 × 10${}^{-4}$	89.7026	0.0012	4
Zip Codes	7.36 × 10${}^{-18}$	2.61 × 10${}^{-36}$	4.96 × 10${}^{-22}$	8.17 × 10${}^{-45}$	0.0787	3.74 × 10${}^{-5}$	74.0513	0.0047	6
Finger Print	5.72 × 10${}^{-18}$	1.19 × 10${}^{-36}$	1.67 × 10${}^{-22}$	2.23 × 10${}^{-45}$	0.2716	1.57 × 10${}^{-4}$	55.952	6.49 × 10${}^{-6}$	0

**Table 4:** Comparative experiment-I separation results.

n	N	VCA		MVSA		N-FINDR		Proposed	
		mErrA	vErrA	mErrA	vErrA	mErrA	vErrA	ErrA	TnMiss
5	10,000	0.0755	7.08 × 10${}^{-5}$	0.0813	6.65 × 10${}^{-5}$	0.0905	4.14 × 10${}^{-5}$	—	100
7	10,000	0.056	9.63 × 10${}^{-6}$	0.0567	2.6 × 10${}^{-5}$	0.0604	7.23 × 10${}^{-6}$	—	100
9	10,000	0.0422	2.96 × 10${}^{-6}$	0.0402	1.13 × 10${}^{-5}$	0.0441	1.34 × 10${}^{-6}$	—	100
11	10,000	0.0333	8.56 × 10${}^{-7}$	0.0314	4.16 × 10${}^{-6}$	0.0342	7.16 × 10${}^{-7}$	—	100
13	10,000	0.0269	3.28 × 10${}^{-7}$	0.0252	1.56 × 10${}^{-6}$	0.0274	3.6 × 10${}^{-7}$	—	100
15	10,000	0.0223	1.52 × 10${}^{-7}$	0.0212	7.53 × 10${}^{-7}$	0.0226	1.8 × 10${}^{-7}$	—	100

**Table 5:** Comparative experiment-II separation results.

n	N	VCA	MVSA	N-FINDR	Proposed
5	10,000	0.0798	0.0872	0.0954	6.43 × $10^{-10}$
7	10,000	0.0573	0.045	0.0658	4.73 × $10^{-10}$
9	10,000	0.0407	0.0309	0.0467	3.77 × $10^{-10}$
11	10,000	0.0321	0.0265	0.0357	0.0012
13	10,000	0.0255	0.0231	0.0284	0.0007
15	10,000	0.0212	0.0197	0.0235	0.0021

**Table 6:** Effect of parameter ρ on the total number of failures.

n	N	ρ = 0	ρ = 0.2	ρ = 0.4	ρ = 0.6	ρ = 0.8	ρ = 1
5	10,000	0	0	0	0	0	0
7	10,000	92	24	13	7	5	4
9	10,000	99	32	16	12	7	6
11	10,000	100	47	27	19	15	10
13	10,000	100	53	26	17	15	13
15	10,000	100	69	45	29	22	18

## References

[1] Joho M., Mathis H., Lambert R.H. Overdetermined blind source separation: Using more sensors than source signals in a noisy mixture. Proceedings of the Independent Component Analysis and Blind Signal Separation.

[2] Winter S., Sawada H., Makino S. (2006). Geometrical interpretation of the PCA subspace approach for overdetermined blind source separation. EURASIP J. Adv. Signal Process..

[3] Bofill P., Zibulevsky M. (2001). Underdetermined blind source separation using sparse representations. Signal Process..

[4] Zhen L., Peng D., Yi Z., Xiang Y., Chen P. (2017). Underdetermined blind source separation using sparse coding. IEEE Trans. Neural Netw. Learn. Syst..

[5] Herault J., Jutten C., Ans B. (1985). Detection de Grandeurs Primitives dans un Message Composite par une Architecture de Calcul Neuromimetique en Apprentissage Non Supervise. 1985—GRETSI—Actes de Colloques.

[6] Syed M., Georgiev P., Pardalos P. (2012). A hierarchical approach for sparse source blind signal separation problem. Comput. Oper. Res..

[7] Hyvärinen A., Karhunen J., Oja E. (2004). Independent Component Analysis.

[8] Amari S.I., Cichocki A., Yang H.H. A new learning algorithm for blind signal separation. Proceedings of the 8th International Conference on Neural Information Processing Systems.

[9] Comon P. (1994). Independent component analysis, a new concept?. Signal Process..

[10] Hyvärinen A. New approximations of differential entropy for independent component analysis and projection pursuit. Proceedings of the 1997 Conference on Advances in Neural Information Processing Systems.

[11] Bell A.J., Sejnowski T.J. (1995). An information-maximization approach to blind separation and blind deconvolution. Neural Comput..

[12] Chai R., Naik G.R., Nguyen T.N., Ling S.H., Tran Y., Craig A., Nguyen H.T. (2017). Driver fatigue classification with independent component by entropy rate bound minimization analysis in an EEG-based system. IEEE J. Biomed. Health Inform..

[13] Naik G.R., Baker K.G., Nguyen H.T. (2015). Dependence independence measure for posterior and anterior EMG sensors used in simple and complex finger flexion movements: Evaluation using SDICA. IEEE J. Biomed. Health Inform..

[14] Naik G.R., Al-Timemy A.H., Nguyen H.T. (2016). Transradial amputee gesture classification using an optimal number of sEMG sensors: An approach using ICA clustering. IEEE Trans. Neural Syst. Rehabil. Eng..

[15] Deslauriers J., Ansado J., Marrelec G., Provost J.S., Joanette Y. (2017). Increase of posterior connectivity in aging within the Ventral Attention Network: A functional connectivity analysis using independent component analysis. Brain Res..

[16] O’Muircheartaigh J., Jbabdi S. (2017). Concurrent white matter bundles and grey matter networks using independent component analysis. NeuroImage.

[17] Hand B.N., Dennis S., Lane A.E. (2017). Latent constructs underlying sensory subtypes in children with autism: A preliminary study. Autism Res..

[18] Arya Y. (2017). AGC performance enrichment of multi-source hydrothermal gas power systems using new optimized FOFPID controller and redox flow batteries. Energy.

[19] Al-Ali A.K.H., Senadji B., Naik G.R. Enhanced forensic speaker verification using multi-run ICA in the presence of environmental noise and reverberation conditions. Proceedings of the 2017 IEEE International Conference on Signal and Image Processing Applications (ICSIPA).

[20] Comon P., Jutten C. (2010). Handbook of Blind Source Separation: Independent Component Analysis and Applications.

[21] Nascimento J.M., Dias J.M. (2005). Does independent component analysis play a role in unmixing hyperspectral data?. IEEE Trans. Geosci. Remote Sens..

[22] Syed M., Georgiev P., Pardalos P. (2015). Robust Physiological Mappings: From Non-Invasive to Invasive. Cybern. Syst. Anal..

[23] Georgiev P., Theis F., Cichocki A., Bakardjian H. (2007). Sparse component analysis: A new tool for data mining. Data Mining Biomedicine.

[24] Naanaa W., Nuzillard J. (2005). Blind source separation of positive and partially correlated data. Signal Process..

[25] Chan T.H., Ma W.K., Chi C.Y., Wang Y. (2008). A convex analysis framework for blind separation of non-negative sources. IEEE Trans. Signal Process..

[26] Chan T.H., Chi C.Y., Huang Y.M., Ma W.K. (2009). A convex analysis-based minimum-volume enclosing simplex algorithm for hyperspectral unmixing. IEEE Trans. Signal Process..

[27] Syed M.N., Georgiev P.G., Pardalos P.M. (2015). Blind Signal Separation Methods in Computational Neuroscience. Modern Electroencephalographic Assessment Techniques: Theory and Applications.

[28] Ma W.K., Bioucas-Dias J.M., Chan T.H., Gillis N., Gader P., Plaza A.J., Ambikapathi A., Chi C.Y. (2014). A signal processing perspective on hyperspectral unmixing: Insights from remote sensing. IEEE Signal Process. Mag..

[29] Bioucas-Dias J.M., Plaza A., Dobigeon N., Parente M., Du Q., Gader P., Chanussot J. (2012). Hyperspectral unmixing overview: Geometrical, statistical, and sparse regression-based approaches. IEEE J. Sel. Top. Appl. Earth Obs. Remote Sens..

[30] Yin P., Sun Y., Xin J. (2016). A geometric blind source separation method based on facet component analysis. Signal Image Video Process..

[31] Lin C.H., Chi C.Y., Wang Y.H., Chan T.H. (2016). A Fast Hyperplane-Based Minimum-Volume Enclosing Simplex Algorithm for Blind Hyperspectral Unmixing. IEEE Trans. Signal Process..

[32] Zhang S., Agathos A., Li J. (2017). Robust Minimum Volume Simplex Analysis for Hyperspectral Unmixing. IEEE Trans. Geosci. Remote Sens..

[33] Naanaa W., Nuzillard J.M. (2017). Extreme direction analysis for blind separation of nonnegative signals. Signal Process..

[34] Sun Y., Ridge C., Del Rio F., Shaka A., Xin J. (2011). Postprocessing and sparse blind source separation of positive and partially overlapped data. Signal Process..

[35] Aharon M., Elad M., Bruckstein A. (2006). On the uniqueness of overcomplete dictionaries, and a practical way to retrieve them. Linear Algebra Appl..

[36] Georgiev P., Theis F., Ralescu A. (2007). Identifiability conditions and subspace clustering in sparse BSS. Independent Component Analysis and Signal Separation.

[37] Drumetz L., Veganzones M.A., Henrot S., Phlypo R., Chanussot J., Jutten C. (2016). Blind hyperspectral unmixing using an Extended Linear Mixing Model to address spectral variability. IEEE Trans. Image Process..

[38] Amini F., Hedayati Y. (2016). Underdetermined blind modal identification of structures by earthquake and ambient vibration measurements via sparse component analysis. J. Sound Vib..

[39] Gribonval R., Schnass K. (2010). Dictionary Identification—Sparse Matrix-Factorization via *l*_1_-Minimization. IEEE Trans. Inf. Theory.

[40] Kreutz-Delgado K., Murray J., Rao B., Engan K., Lee T., Sejnowski T. (2003). Dictionary learning algorithms for sparse representation. Neural Comput..

[41] Nascimento J.M., Bioucas-Dias J.M. Blind hyperspectral unmixing. Proceedings of the SPIE Conference on Image and Signal Processing for Remote Sensing XIII.

[42] Duarte L.T., Moussaoui S., Jutten C. (2014). Source separation in chemical analysis: Recent achievements and perspectives. IEEE Signal Process. Mag..

[43] Sun Y., Xin J. (2012). Nonnegative Sparse Blind Source Separation for NMR Spectroscopy by Data Clustering, Model Reduction, and *ℓ*_1_ Minimization. SIAM J. Imag. Sci..

[44] Winter M.E. N-FINDR: An algorithm for fast autonomous spectral end-member determination in hyperspectral data. Proceedings of the Imaging Spectrometry V.

[45] Nascimento J.M., Dias J.M. (2005). Vertex component analysis: A fast algorithm to unmix hyperspectral data. IEEE Trans. Geosci. Remote Sens..

[46] Santamaria I., Pokharel P.P., Principe J.C. (2006). Generalized correlation function: Definition, properties, and application to blind equalization. IEEE Trans. Signal Process..

[47] Liu W., Pokharel P.P., Príncipe J.C. (2007). Correntropy: Properties and applications in non-Gaussian signal processing. IEEE Trans. Signal Process..

[48] Singh A., Principe J.C. Using correntropy as a cost function in linear adaptive filters. Proceedings of the 2009 International Joint Conference on Neural Networks.

[49] Zhao S., Chen B., Principe J.C. Kernel adaptive filtering with maximum correntropy criterion. Proceedings of the 2011 International Joint Conference on Neural Networks.

[50] Chen B., Xing L., Liang J., Zheng N., Principe J.C. (2014). Steady-state mean-square error analysis for adaptive filtering under the maximum correntropy criterion. IEEE Signal Process. Lett..

[51] Chen B., Xing L., Zhao H., Zheng N., Principe J.C. (2016). Generalized correntropy for robust adaptive filtering. IEEE Trans. Signal Process..

[52] Chen B., Liu X., Zhao H., Principe J.C. (2017). Maximum correntropy Kalman filter. Automatica.

[53] Syed M.N., Pardalos P.M., Principe J.C. (2014). On the optimization properties of the correntropic loss function in data analysis. Optim. Lett..

[54] Kuhn H.W. (2010). The hungarian metRhod for the assignment problem. 50 Years of Integer Programming 1958–2008.

[55] Cplex I. (2011). User-Manual CPLEX.

[56] Li J., Bioucas-Dias J.M. Minimum volume simplex analysis: A fast algorithm to unmix hyperspectral data. Proceedings of the 2008 IEEE International Geoscience and Remote Sensing Symposium.

[57] Terlaky T. (2013). Interior Point Methods of Mathematical Programming.

[58] Dantzig G.B., Thapa M.N. (2006). Linear Programming 2: Theory and Extensions.

[59] Andersen E.D., Andersen K.D. (1995). Presolving in linear programming. Math. Program..

